# Development and Optimization of Acriflavine-Loaded Polycaprolactone Nanoparticles Using Box–Behnken Design for Burn Wound Healing Applications

**DOI:** 10.3390/polym14010101

**Published:** 2021-12-28

**Authors:** Touseef Nawaz, Muhammad Iqbal, Barkat Ali Khan, Asif Nawaz, Talib Hussain, Khaled M. Hosny, Walaa A. Abualsunun, Waleed Y. Rizg

**Affiliations:** 1 Faculty of Pharmacy, Gomal University, D. I. Khan 29050, Pakistan; touseefktk@gmail.com (T.N.); barkat.khan@gu.edu.pk (B.A.K.); asifnawaz@gu.edu.pk (A.N.); 2 Institute of Pharmaceutical Sciences, University of Veterinary and Animal Sciences, Lahore 54000, Pakistan; talibhussain@uvas.edu.pk; 3 Department of Pharmaceutics, Faculty of Pharmacy, King Abdulaziz University, Jeddah 21577, Saudi Arabia; kmhomar@kau.edu.sa (K.M.H.); dr.iqbal.graduate@gmail.com (W.A.A.); wrizq@kau.edu.sa (W.Y.R.)

**Keywords:** burn wound, acriflavine, polycaprolactone, Box–Behnken design, nanoparticles

## Abstract

Nanoparticles are used increasingly for the treatment of different disorders, including burn wounds of the skin, due to their important role in wound healing. In this study, acriflavine-loaded poly (ε-caprolactone) nanoparticles (ACR-PCL-NPs) were prepared using a double-emulsion solvent evaporation method. All the formulations were prepared and optimized by using a Box–Behnken design. Formulations were evaluated for the effect of independent variables, i.e., poly (ε-caprolactone) (PCL) amount (X1), stirring speed of external phase (X2), and polyvinyl alcohol (PVA) concentration (X3), on the formulation-dependent variables (particle size, polydispersity index (PDI), and encapsulation efficiency) of ACR-PCL-NPs. The zeta potential, PDI, particle size, and encapsulation efficiency of optimized ACR-PCL-NPs were found to be −3.98 ± 1.58 mV, 0.270 ± 0.19, 469.2 ± 5.6 nm, and 71.9 ± 5.32%, respectively. The independent variables were found to be in excellent correlation with the dependent variables. The release of acriflavine from optimized ACR-PCL-NPs was in biphasic style with the initial burst release, followed by a slow release for up to 24 h of the in vitro study. Morphological studies of optimized ACR-PCL-NPs revealed the smooth surfaces and spherical shapes of the particles. Thermal and FTIR analyses revealed the drug–polymer compatibility of ACR-PCL-NPs. The drug-treated group showed significant re-epithelialization, as compared to the controlled group.

## 1. Introduction

Each year, acute thermal burns and injuries affect nearly half a million Americans who require medical treatment, of which approximately 40,000 need hospitalization. In the past four decades, the survival rate of burn wounds has improved amazingly; this is attributed to new treatment strategies for burn wounds, advancements and improvements in burn-care units, research on burn-wound management, and artificial skin grafting [1]. All these improvements helped us in controlling burn mortalities. Nearly 80% of burns are due to wet sources (scalds) and dry sources (flames and fire), and can be distinguished based on burn depth. A thermal injury greater than 20% of the total body surface area or greater leads to burn shock. Burn shock is characterized by fluids and protein movement from intravascular to interstitial space, increased capillary permeability, a hydrostatic pressure increase in the microvasculature, a decrease in cardiac output, and a decrease in body fluids, which leads to hypovolemia [2,3].

Recently, nanoparticles received enormous attention due to their small particle size and high surface area, sustained action, and targeted drug-delivery properties [4]. Nanoparticles are advantageous over liposomes, having properties to overcome liposome limitations such as a low entrapment efficiency, instability, and drug leakage [5]. Nanoparticle applications are gaining popularity in different fields, including pharmaceuticals, cosmetics, agriculture, and the food industry [6]. Different polymeric biomaterials can be used for the preparation of nanocarriers for drug-delivery systems, and they can be categorized into two classes, i.e., biodegradable and non-biodegradable biomaterials. Their discovery made a breakthrough in different fields, such as gene therapy, tissue engineering, controlled drug-delivery systems, and regenerative medicines. Polymeric biomaterials can be natural as well as synthetic. The naturally produced biopolymers are proteins and polysaccharides, whereas synthetically produced biopolymers are aliphatic polyesters and polyphosphoester (PPE). Synthetically produced biopolymers have minimum immunogenicity, as compared to naturally occurring biopolymers [7]. Another advantage of using synthetic biopolymers is their modification ability for a specific function [8]. Among various biodegradable polymers, polycaprolactone (PCL), polylactide, and poly (lactic-co-glycolic acid) are commonly used in drug-delivery systems. These are semi-crystalline polyesters with low melting points (60 °C) and a glass-transition temperature that allows for their easy processing. PCL is known for its versatility in encapsulating a wide variety of drugs. It is biocompatible, biodegradable, and non-toxic in nature, having therapeutic carrier potential capabilities [9]. It is insoluble in alcohols and water but soluble in organochloride compounds (such as chloroform, DCM, etc.) and aromatic solvents. PCL has the ability to form a blend with other polymers, making their physicochemical properties highly versatile [10]. The double-emulsion solvent evaporation method can be used for the preparation of PCL nanocarriers; this method can be used for the encapsulation of both lipophilic as well as hydrophilic drugs simultaneously. Thus, by utilizing the potential application of this method, the co-delivery of two different nature drugs would be possible [11].

Acriflavine chemical names are euflavine, xanth acridine, diagrid, isravin, etc., and they have a molecular formula of C_2_7H_2_5CIN_6_. Its IUPAC name is Acridine-3,6-diamine,10-methyl acridine-10-ium-3,6-diamine, Chloride [12]. In powder form, acriflavine is deep orange to brownish in color. The melting point of acriflavine is 179–181 °C and the boiling point is 612 °C. It is soluble in water (0.33 g/mL) and insoluble in polar organic solvents [13]. Acriflavine is also known for its fluorescent nature in confocal laser scanning microscopy studies [14]. It can also be used to inhibit vascularization, tumor growth factors, and HIF-I dimerization [15,16]. It can be used against E-coli and drug-resistant staphylococcus aureus [17]. Acriflavine belongs to the aminoacridine class of drugs, which has good antiseptic capabilities and is used as a topical antiseptic [18,19].

The aim of this study was to prepare and optimize acriflavine-loaded PCL nanoparticles for the treatment of burn wounds. Design Expert^®^ version 11 (State-Ease Inc., Minneapolis, MN, USA) was used to optimize the formulations of ACR-PCL-NPs. Optimized NPs were further processed for a characterization study, including zeta potential, PDI, hydrodynamic particle size, particle morphology, FTIR, DSC, and in vitro drug-release studies using Franz diffusion cells.

## 2. Materials and Methods

### 2.1. Materials

Acriflavine was kindly donated by Pharmawise Labs, Lahore, Pakistan. Polycaprolactone (PCl) (14,000 g/mol), Polyvinyl alcohol (PVA) (31,000 g/mol), and Dichloromethane (DCM) were purchased from Sigma–Aldrich chemicals, Saint Louis, MO, USA. All chemicals used in the experimentation were of analytical grade. Distilled water was obtained from our laboratory’s distillation plant (laboratory water still, Stuart Equipment, Stone, UK).

### 2.2. Preparation of Acriflavine Loaded Nanoparticles

The double-emulsion solvent evaporation method was adopted for the preparation of the ACR-PCL-NPs, as described by Ibraheem et al. [20]. The formulations were synthesized according to the parameters composition (independent variables) given by the Box–Behnken designas given in Table 1. All 15 formulations were prepared by a two-step process; in the first step, 0.5 mL deionized water was used as the inner aqueous phase (W1), and it was homogenized at 3000 rpm for 3 min with the oil phase (different amounts of PCL were dissolved in 4 mL dichloromethane) to form a water-in-oil emulsion (primary emulsion), while in the second step, the first emulsion was added to the external aqueous phase (W2) (60 mL) containing different concentrations (0.3%, 0.5%, and 0.7%) of PVA and mixed properly at different stirring speeds (8000 rpm, 10,000 rpm, and 12,000 rpm) to form a double emulsion by using a homogenizer (DAIHAN Scientific, Wonju, HG-15A, Korea). The outer aqueous phase (W2) was prepared by adding polyvinyl alcohol (PVA) (3 g, 5 g, and 7 g) to 1 L of deionized water to form the PVA solution. In order to get a clear PVA solution, it was stirred for 40 min using a magnetic stirrer at 60 °C. In the second step, the droplets of the primary emulsion solidify in the presence of the external phase, which leads to the formation of suspended polymeric particles. Finally, the organic solvent was eliminated with the help of a rotary evaporator. In addition, the particles were centrifuged at 10,000 rpm for 10 min, and the particles obtained after centrifugation were re-suspended in deionized water; this process was performed in triplicate. After washing, the recovered nanoparticles were collected and freeze-dried. The freeze-dried nanoparticles were kept at 4 °C and were characterized physicochemically [21].

### 2.3. Design for Optimization

Optimization is a robust approach for an accurate and precise formulation in all aspects. Design Expert (Design Expert^®^ version 11.0.5.0, State Ease Incorporation, Minneapolis, MN, USA) was used for the optimization of the formulation using a Box–Behnken design with three levels (low −1, medium 0, high +1) as shown in Table 2. In this study, three independent factors, including polymer concentration (X1), stirring speed (X2), and surfactant concentration (X3) was used over three dependent responses (Table 1), i.e., particle size (Y1), polydispersity index (Y2), and entrapment efficiency (Y3), for the optimization of the formulations. As shown in the Table 3, a total of 15 experimental runs were executed to acquire the optimized formulation with the statistical data, which were further processed for physicochemical characterization. The optimized formulation was selected on the basis of factor desirability over responses [22].

### 2.4. Characterization

#### 2.4.1. Polydispersity, Particle Size, and Zeta Potential

The prepared formulations were subjected to particle size, polydispersity, and zeta potential analyses using Zetasizer (Nano ZS, Malvern, Malvern WR14 1XZ, UK). About 1 mM NaCl was added prior to the zeta analysis to produce isotonic stability. A 100-fold dilution of the samples was performed to avoid multiple scattering using deionized water. A scattering angle of 90° at 25 ± 1 °C for each sample was used for characterization [23].

#### 2.4.2. Encapsulation Efficiency (EE)

The %EE of optimized acriflavine nanoparticle formulations were measured using slightly modified indirect methods. A specified amount of particulate suspension from each formulation was centrifuged using centrifugation tubes at 15,000 rpm for 30 min at 4 °C. The supernatant layer was collected and analyzed for free drug content at 416 nm by adding 2.5 mL of the sample in the spectrophotometer covit, and the %EE of the optimized formulation was measured using the following formula:

(1) $$\mathrm{Encapsulation}\;\mathrm{efficiency}\;\left(\%\mathrm{EE}\right)=\frac{\mathrm{Total}\;\mathrm{acriflavine}-\mathrm{acriflavine}\;\mathrm{in}\;\mathrm{supernatent}}{\mathrm{Total}\;\mathrm{acriflavine}}\times 100$$

A standard curve was produced by preparing a series of acriflavine dilutions in deionized water. The results of all formulations were quantified using a standard curve [22].

#### 2.4.3. Particles Morphology

The optimized ACR-PCL-NPs’ morphology was assessed using a scanning electron microscope (SEM). Before the SEM analysis, the optimized ACR-PCL-NPs were lyophilized using a freeze-dryer (Biobase, Shandong, China). Lyophilized nanoparticles were mounted on aluminum stubs supported by adhesive tape. The SEM (Carl Zeiss Inc., Oberkochen, Germany) was operated to visualize the morphology of the ACR-PCL-NPs under high vacuum at 10 KV accelerated voltage [24,25].

#### 2.4.4. Differential Scanning Calorimetry (DSC)

DSCs of pure acriflavine, PCL, a physical mixture of acriflavine and PCL, and ACR-PCL-NPs were performed to assess any change in physical state (PerkinElmer, Pyres 6.0 DSC, Waltham, MA, USA). A sample of 3 mg was used in a heating pan on a heating range of 40–300 °C and with a heating rate of 10 °C/minute, using nitrogen gas with a 20 mL/minute flow [26,27].

#### 2.4.5. Attenuated Total Reflectance Fourier Transform Infrared (ATR-FTIR) Spectroscopy

ATR-FTIR was used to determine drug-interaction with components and the chemical composition of each ingredient in the formulation. Pure acriflavine, PCL, and ACR-PCL-NPs spectra were obtained using ATR-FTIR fitted with an ATR sampling cell (Thermo Scientific, Waltham, MA, USA). A resolution of 4 cm^−1^ was used to obtain FTIR spectra between 4000 and 400 cm^−1^. Two scans of each sample were performed and spectra were obtained using OPUS 5 software.

### 2.5. In Vitro Release Study

An in vitro release study of optimized ACR-PCL-NPs was performed using the Franz diffusion cell method. A sample of 1 mL was applied on a cellulose membrane and it was placed between the donor and receptor compartments, and a phosphate buffer of pH 7.4 was added to the receptor compartment and was stirred at 250 rpm using a magnetic bead with the temperature maintained at 32 ± 1 °C. A sample of 1 mL was collected at different time intervals (0.5 h, 1 h, 1.5 h, 2 h, 4 h, 8 h, 12 h, 16 h, 20 h, and 24 h) and the buffer volume was replaced with 1 mL fresh dissolution media (pH 7.4). Collected samples were diluted and the acriflavine content was determined using a UV-visible spectrophotometer at 416 nm. Furthermore, kinetic release models were applied to the acriflavine release data from the optimized ACR-PCL-NPs to estimate the acriflavine release mechanism [28,29].

### 2.6. Histological Examination

All the experiments involving rats were conducted in accordance with NIH (USA) Care and Use of Lab Animals (NIH, 25 June 1985). The optimized formulation was tested in vivo for wound healing activity. For this purpose, 24 male rats were purchased from the National Institute of Health, weighing 250 ± 10 g. They were housed in separate cages and provided with free access to food and water for 7 days. Then, they were divided into 3 equal groups. Group 1 was a control group, group 2 was treated with an acriflavine-containing formulation, and group 3 was treated with a marketed drug (1% acriflavine). In order to anesthetize the lab animals, rats were injected intramuscularly with ketamine (40 mg/kg of body weight) and xylazine (5 mg/kg body weight). After 14 days of this treatment, rats were sacrificed to excise and collect the burn-wound sites for hematoxylin and eosin staining (H and E staining). The excised wound was washed using normal saline water and it was formalin-fixed. An approximately 3–5-µm thick section of the excised wound was stained using H and E staining to be photographed under a light microscope at 5× and 40× magnification [30]. Moreover, quantitative percent re-epithelialization was determined using the following formula:

(2) % Re−epithhelialiazation=B −C B ×100

where B is a re-epithelialized skin area and C is an unclosed wound [31].

Statistical analysis was performed and the data are expressed as mean ± SD from three separate observations. For different content assays, a one way ANOVA test (*p* < 0.05) was used to analyze the differences among EC50. A probability of *p* < 0.05 was considered as significant.

## 3. Results and Discussion

### 3.1. Optimization of ACR-PCL-NPs

A Box–Behnken design was adopted for the ACR-PCL-NPs’ optimization. The effects of the independent variables were investigated over each dependent variable using a contour plot and a 3D response surface plot. A polynomial equation was used to check the combined and individual effect of each factor on each response using Design Expert software. The quadratic effect was best applied on all factors because it offers the maximum effect both individually and in combination. The Design software managed the analysis of variance (ANOVA) of individual responses, and the results indicate the model-fitting for the data sets, as shown in Table 4 (linear, 2FI, quadratic). The factors such as polymer concentration (X1), stirring speed (X2), and surfactant concentration (X3) were evaluated at three levels (low −1, medium 0, high +1) to formulate ACR-PCL-NPs.

The selection of optimized ACR-PCL-NPs was made on the basis of specific criteria, i.e., the small size of the nanoparticles, by Design Expert.

A total of 15 formulations with three central points were fed into the Design Expert software. After analysis, the particle size (Y1) of formulation F1 was found to be 302.4 nm, which was the smallest of all 15 formulations, as shown in Table 3. Similarly, the particle size of formulation F14 was 780.4 nm, the highest of all formulations. F1 had the lowest, at 0.202% PDI (Y2), while F13 has the highest, at 0.349% PDI. The encapsulation efficiency (Y3) of formulation F2 was the lowest, i.e., 67.7%, while formulation F15 was the highest, at 81.8%, as shown in Table 3. The coefficient of correlation (R^2^) values were in the range of 0.8699 to 0.9864, which shows the highest coherence of fit data with high value PRESS, as described in Table 4.

### 3.2. Particle Size, Zeta Potential, and PDI

**Figure 1:**
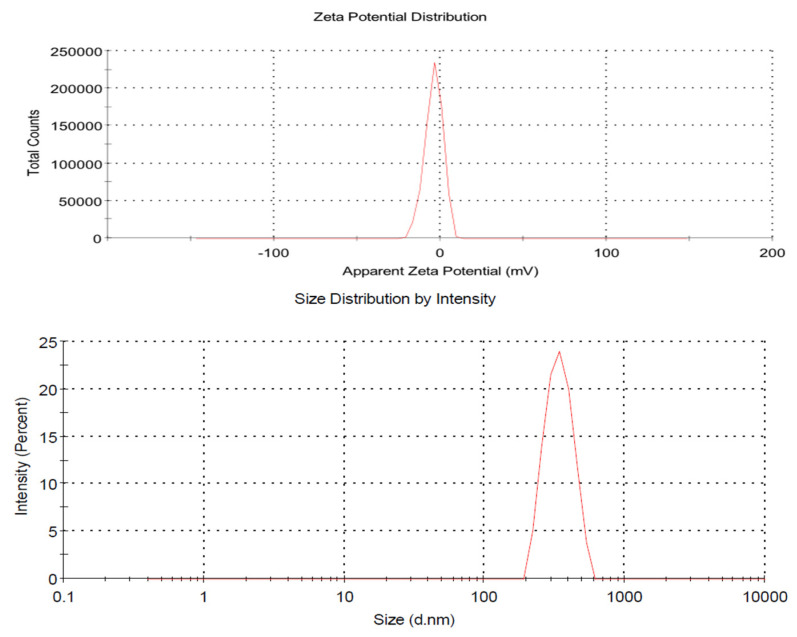
Average particle size distribution and zeta potential graphs of optimized ACR-PCL-NPs.

The hydrodynamic particle size of ACR-PCL-NPs was examined. The obtained values of the ACR-PCL-NPs were the average of three independent measurements. The particle size and PDI of the optimized ACR-PCL-NPs were 469.2 ± 5.6 nm and 0.270 ± 0.19, respectively. A small particle size means a large surface area for release, and a low PDI ensures the homogeneity of the particles [22]. Zeta potential is an analytical technique used to measure the surface charge of NPs in colloidal dispersions. The zeta potential magnitude provides signs of colloidal stability. NPs with zeta potential values between >+25 and <−25 mV have high degrees of stability; low zeta potential values may lead to coagulation, flocculation, and aggregation due to van der Waals forces [32,33]. The zeta potential of ACR-PCL-NPs were found between −3.75 and −7.96 mV, which were considered to be near zero (Figure 1). The results demonstrate no considerable variation in the zeta potentials of the formulations, which is associated with the uncharged chemical nature of PCL, as already reported in the literature [34,35].

#### 3.2.1. Effect of Independent Variables on Particle Size of ACR-PCL-NPs (Y1)

**Figure 2:**
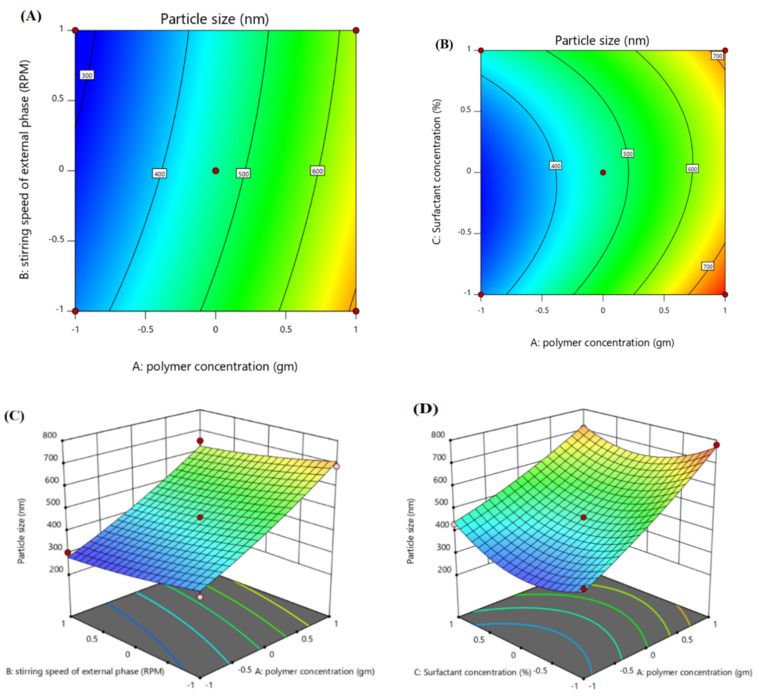
Contour surface plots showing the effect of independent variables (factors) on the particle size (Y1) of optimized ACR-PCL-NPs formulation. (**A**) Impact of polymer concentration and stirring speed on particle size, (**B**) impact of polymer concentration and surfactant concentration on particle size) and 3D plots, (**C**) impact of polymer concentration and stirring speed on particle size, (**D**) impact of polymer concentration and surfactant concentration on particle size.

Contour plots and 3D surface plots were used to check the effect of independent variables on the ACR-PCL-NPs, as shown in Figure 2. The PCL concentration (X1) had a positive effect on the particle size of nanoparticles. An increase in PCL concentration, from 1 g to 2 g (F1 and F10), led to an increase in particle size, from 302.4 nm to 730 nm of ACR-PCL-NPs, as shown in the Table 3. Our results are in accordance with Lepeltier and co-workers’ study, where they state that an increase in polymer concentration causes an increase in the nanoparticles’ size [36,37]. The stirring speed of the secondary emulsion (X2) had a negative impact on the size of the prepared nanoparticles. As we increased the stirring speed of the external phase from 8000 rpm (F11) to 12,000 rpm (F5), a decrease in particle size from 670 nm to 499 nm was observed, which was similar to what was demonstrated by Ibraheem et al. [20]. This decrease in particle size may be due to the provision of high levels of energy to the particulate system, as high stirring speeds cause the dismantling of large droplets of the second emulsion into small droplets [38]. As we increased the stirring speed of the formulations, a gradual decrease in the particle size of the formulations was detected. PVA should be used in optimum concentrations; low concentrations of PVA (X3) lead to coalescence, while high concentrations of PVA can cause clumping of the nanoparticles [39]. The combined effect of both polymer concentration (X1) and surfactant concentration (X3) was positive for the particle size, and the effect of stirring speed in the external phase (X2) was negative on the particle size.

Particle Size (Y1):

(3) $$462.47+181.81X1-33.09X2+24.40X3+20.13X1X2+4.25X1X3\;-19.05X2X3+31.03X1^{2}+22.43X2^{2}+81.75X3^{2}$$

The polynomial equation (Equation (1)) indicates that X1 and X3 have positive effects on the particle size (Y1) of the ACR-PCL-NPs (*p* < 0.0001). The model’s F-value, 16.28, and *p*-value, 0.0034, suggests the model’s significance. The lowest *p*-value and highest F-value indicates the best fit of the model. The Prob F-value was 0.005. All the independent variables showed a significant effect on the ACR-PCL-NPs. The R^2^ and adjusted R^2^ values were in close range, which shows the model’s robust credibility.

#### 3.2.2. Effect of Independent Variables on PDI (Y2)

**Figure 3:**
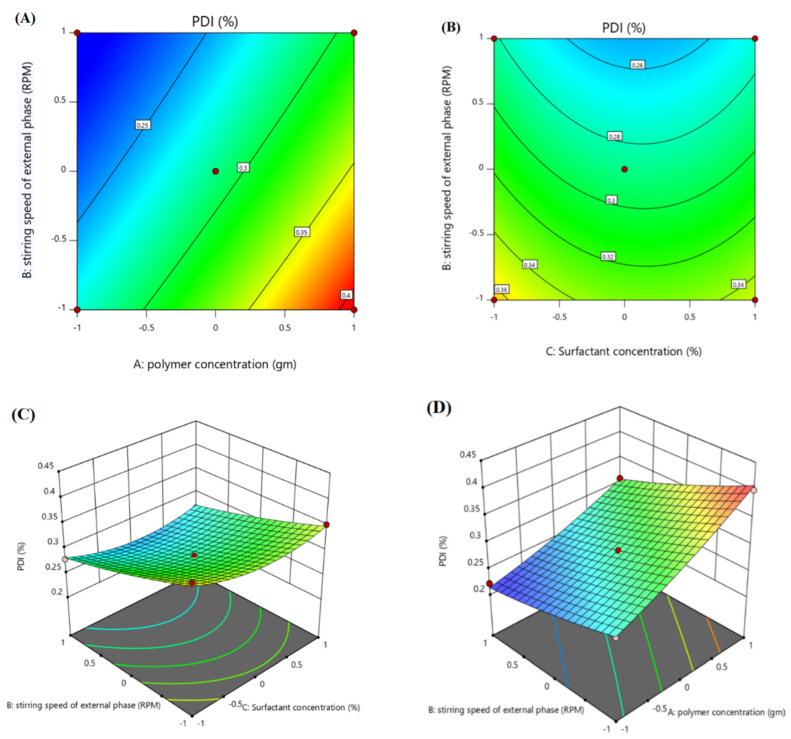
Contour surface plots showing the effect of independent variables (factors) on the polydispersity index (Y2) of the optimized ACR-PCL-NPs. (**A**) Impact of polymer concentration and stirring speed on PDI, (**B**) impact of stirring speed and surfactant concentration on PDI and 3D plots, (**C**) impact of stirring speed and surfactant concentration on the PDI, (**D**) impact of polymer concentration and stirring speed on the PDI.

The polymer concentration (X1) has shown a high positive impact on the PDI of the formulations. The higher the PCL concentration that was used, the higher the PDI that was observed, as shown in Table 3. The stirring speed of the external phase (X2) had a slightly negative impact on the PDI, and this was in accordance with Scholz et al.’s findings [40]—that an increase in stirring speed can decrease both the particle size and PDI of the system. Polymer concentration was the prominent factor in determining the formulation of the PDI [41]. The surfactant concentration (X3) had a very slightly negative impact on the PDI of a formulation, as shown in Table 3 and Figure 3. F13, with a PVA concentration of 0.3%, and F14, with a PVA concentration of 0.7%, had 0.399 and 0.363 PDI, respectively, which is in agreement with the work of Tefas et al. [42], where they stated that PVA has a negative effect on the PDI of the formulation when used as a surfactant. The F-value of the model was 40.28, which made the model significant. The *p*-value 0.0004 indicates only a 0.041% chance of noise to occur in the model.

Polydispersity Index (Y2):

(4) $$0.28803+0.0574X1-0.0401X2-0.0068X3-0.0105X1X2-0.0133X1X3\;+0.0018X2X3+0.0076X1^{2}-0.0051X2^{2}+0.0238X3^{2}$$

The higher F-value (40.28) and the lower *p*-value (0.0004) suggest that the model is significant and stable. The R^2^ value and the adjusted R^2^ value were 0.9864 and 0.9619, respectively. The model significance is a sign of a model with low noise.

#### 3.2.3. Effect of Independent Variables on Entrapment Efficiency (%EE) (Y3)

**Figure 4:**
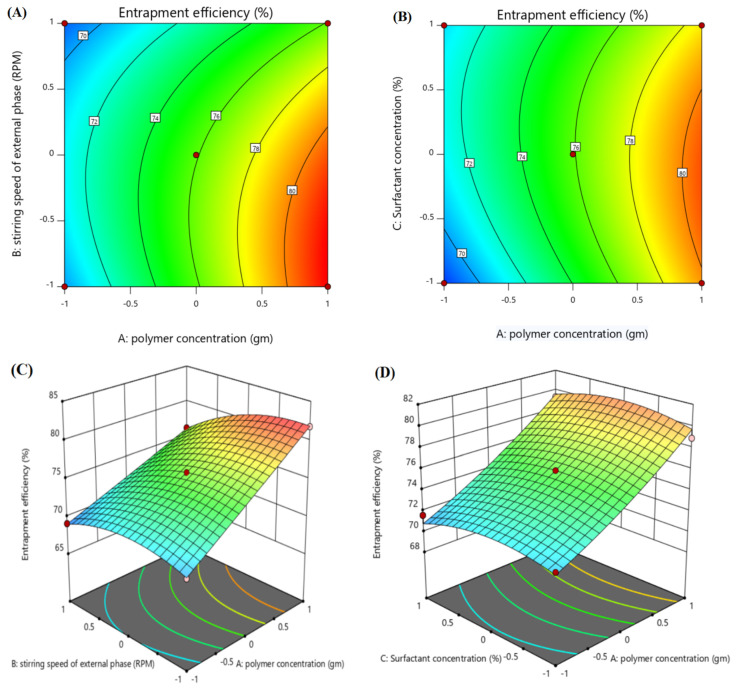
Contour surface plots showing the effect of independent variables (factors) on the entrapment efficiency (Y3) of optimized ACR-PCL-NPs. (**A**) impact of polymer concentration and stirring speed on entrapment efficiency, (**B**) impact of polymer concentration and surfactant concentration on entrapment efficiency and 3D plots, (**C**) impact of stirring speed and polymer concentration on entrapment efficiency, (**D**) impact of polymer concentration and surfactant concentration on entrapment efficiency.

The effect of different factors over %EE (Y3) can be seen in the contour plot and 3D surface plot, as given in Figure 4. The polynomial equation (Equation (4)) showed the positive impact of polymer concentration (X1) and surfactant concentration (X3) on %EE, as demonstrated by Ibraheem et al. [20,36]. Formulations F2 and F13, with %EE of 67.7 and 78.9, respectively, have polymer concentrations of 1g and 2 g, respectively. An increase in polymer concentration had a direct influence on %EE, i.e., increasing the % EE [38]. Similarly, the surfactant concentration (X3) had a positive influence on the % EE. An increase in PVA concentration (X3) will lead to an increase in %EE, because PVA has the tendency to encapsulate the drug in the matrix [43]. The stirring speed (X2) showed a slightly negative impact on the %EE. An increase in stirring speed (X2) led to a decrease in %EE, which is accordance with Bozena et al.’s work, where they stated that increases in stirring speed will decrease the drug content of particles [44]. Similarly, JK Patel’s study also showed a decrease of %EE with an increase of stirring speed, which is an accordance with our results [45].

Entrapment Efficiency (Y3):

(5) $$75.57+4.75X1-1.66X2+0.2875X3-1.22X1X2+0.8750X1X3\;-0.25-X2X3+0.2167X1^{2}-1.56X2^{2}-1.41X3^{2}$$

The polynomial equation (Equation (5)) suggests the positive effect of both PCL (X1) and PVA (X3) on %EE, and the negative impact of stirring speed (X2) on %EE (*p* < 0.001). The model’s F-value, 24.06, suggests the model is significant. The high F-value and low *p*-value (0.0013) delineate the fitness and reliability of the model. The R^2^ and adjusted R^2^ value were 0.9774 and 0.9368, respectively, which are close to each other. The significance and low noise in the model enhances the credibility of the model.

#### 3.2.4. Preparation of Optimized ACR-PCL-NPs

Optimized ACR-PCL-NPs were prepared using same method as followed for all 15 trial formulations. The optimized formulation was formulated on the basis of values given by the Design Expert software after the analysis of 15 trial formulations for independent variables. Optimization was based on the small particle size, low polydispersity index, and high entrapment efficiency. After the analysis of the trial formulations, a formulation with a desirability of 1 was selected as the optimized formulation, having all the above-discussed properties. The software provided actual values for the independent and predicted values of the dependent variables of the optimized formulation. The actual values calculated after optimization for the PCL concentration (X1), the stirring speed of the external phase (X2), and the PVA concentration (X3) were 1.5g, 12,000 rpm, and 0.5%, respectively. The predicted values of the responses for optimized formulation were 451.9 nm for Y1, 0.253 for Y2, and 72.34% for Y3. When the optimized formulation of ACR-PCL-NPs was prepared using actual values of independent variables, the responses’ actual values were 469.2 ± 5.6 nm for Y1, 0.270 ± 0.19 for Y2, and 71.9 ± 5.32% for Y3. The responses’ actual values were close to the predicted values. The optimized ACR-PCL-NPs were processed for characterization, for an in vitro release study, and for a kinetic model-fitting study.

#### 3.2.5. Surface Morphology

**Figure 5:**
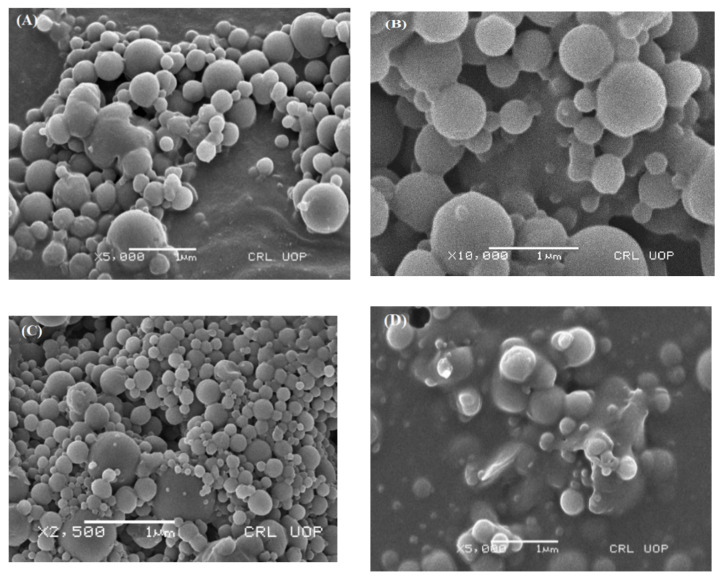
Scanning electron microscope (SEM) images of the optimized ACR-PCL-NPs. (**A**) 5000× magnification, (**B**) 10,000× magnification, (**C**) 2500× magnification), and (**D**) 5000× magnification.

A scanning electron microscope (SEM) was used for the morphological analysis of the ACR-PCL-NPs prepared by using the double-emulsion solvent evaporation method. The surface texture, shape, inter particulate bridging, and smoothness of the ACR-PCL-NPs were evaluated. The SEM images show that the ACR-PCL-NPs were smooth on the surface with a spherical shape. The smoothness of the ACR-PCL-NPs’ surface supports the assumption that encapsulated active drug-release may be due to matrix erosion [46]. Additionally, bridging between particles can be seen in SEM images (Figure 5), and these bridges may be associated with the sticky nature of the PVA used in these formulations. Being sticky in nature, it is very difficult to completely remove the PVA, even after particle washing [47].

#### 3.2.6. Differential Scanning Calorimetry

**Figure 6:**
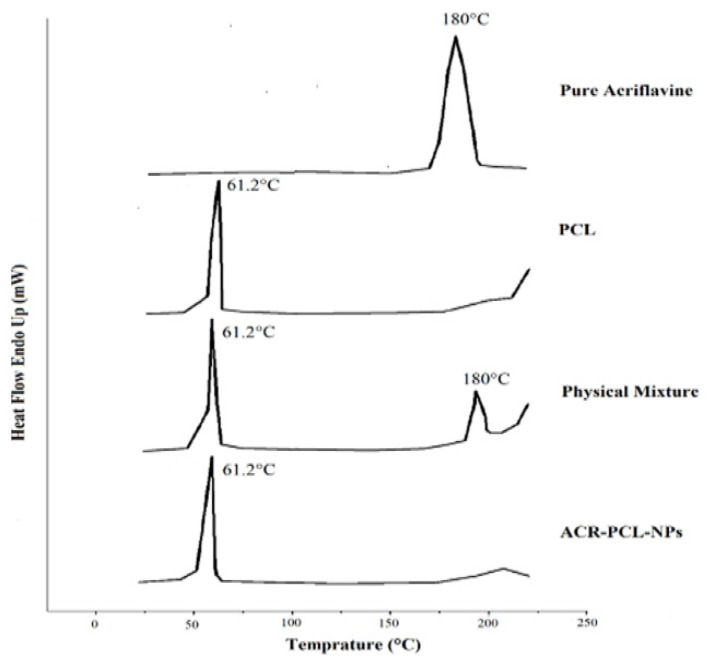
DSC thermogram of acriflavine, PCL, a physical mixture of PCL and acriflavine, and optimized ACR-PCL-NPs.

Figure 6 represents the DSC thermograms of pure acriflavine, PCL, physical mixtures of PCL and acriflavine, and the ACR-PCL-NPs. The DSC thermogram of pure acriflavine represents a sharp endothermic peak at 180 °C, which shows the crystalline nature of the acriflavine. The PCL DSC thermogram showed a sharp peak at 61.2 °C. The physical mixture of acriflavine and PCL showed two distinctive peaks, one at 61.2 °C for PCL, and another at 180 °C for acriflavine. However, the peak of acriflavine in the ARR-PCL-NPs was absent because of the encapsulation of acriflavine in the PCL matrix in an amorphous form [5,48]. A single peak, at 61.2 °C, was observed in the ACR-PCL-NPs’ thermogram. The melting temperature (Tm) of PCL and ACR-PCL-NPs remained the same (61.2 °C), as shown in Figure 6. The degree of crystallinity (Xc) of the ACR-PCL-NPs decreased (PCL = 93% and ACR-PCL-NPs = 75.5%). The enthalpy of fusion (Δ Hm) of PCL and ACR-PCL-NPs were 126.5 J/g and 103 J/g, respectively. The decrease in Xc and ΔHm in the ACR-PCL-NPs was due to a lack of homogenous distribution and structure uniformity [49].

#### 3.2.7. FTIR Analysis

**Figure 7:**
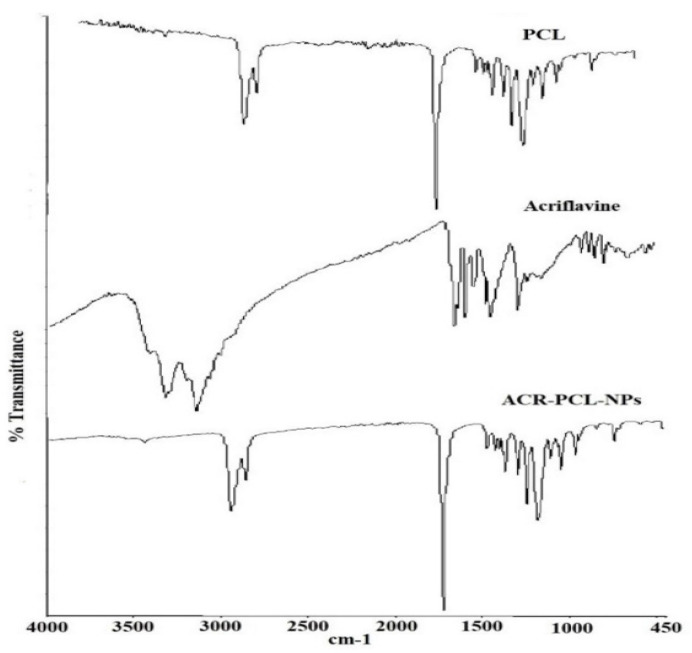
FTIR spectra of acriflavine, pure PCL, and prepared nanoparticles (ACR-PCL-NPs).

Figure 7 represents the FTIR spectra of acriflavine, PCL, and the ACR-PCL-NPs. The characteristic peaks of acriflavine were found at 3207 cm^−1^, which indicates the stretching of symmetric C–NH, at 1324 cm^−1^, which indicates the asymmetric C–N stretching vibrations, at 1130 cm^−1^, which was due to C–H stretching, at 1383 cm^−1^, which was for characteristic cyclic C–N–C vibrations, and at 1171 cm^−1^, which was for the acriflavine CH_3_ group. The CH–CH_3_ group can be confirmed by the peak at 968 cm^−1^; the 929.81-cm^−1^ peak indicates CH_3_ vibrations. PCL is an aliphatic polymer that shows characteristic peaks between 2860–3000 cm^−1^ due to C–H stretching. The 1719-cm^−1^ peak was due to carbonyl group C=O stretching vibrations. The 1044-cm^−1^-1298-cm^−1^ peaks were due to saturated-ester C–O stretching. The 735-cm^−1^ peak represents C–H bending vibrations. The peaks of PCL and ACR-PCL-NPs were similar in structure; only the decrease in the peak of the ACR-PCL-NPs, from 1723 cm^−1^ to 1721 cm^−1^, was due to C=O stretching of unsaturated ester. The peaks of the ACR-PCL-NPs were not clearly observed in the formulation FTIR spectra. This might be due to the presence of acriflavine in molecular dispersion in a polymer matrix [5]. The FTIR spectra do not show any potential interactions or chemical incompatibilities between acriflavine and PCL in the nanoparticles.

### 3.3. In Vitro Release Study

**Figure 8:**
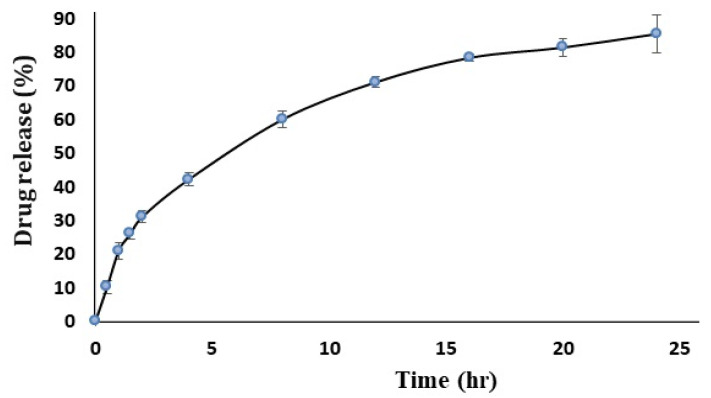
In vitro drug release profile of optimized ACR-PCL-NPs.

The solution of acriflavine showed a 96.5 ± 2.5% release in 1 h, while the optimized ACR-PCL-NPs showed 85.43 ± 5.38% in 24 h. The ACR-PCL-NPs showed a burst release followed by a sustained release, revealing a biphasic release profile (Figure 8). The first burst release was due to the surface drug on ACR-PCL-NPs, and the controlled release was due to the diffusion of acriflavine from the NPs into the release medium [50]. The acriflavine diffusion from the ACR-PCL-NPs was due to its low molecular weight and high solubility in the dissolution medium [51]. The core drug diffuses much more slowly, due to a longer diffusion path. The sustained release was favorable for a prolonged therapeutic effect.

**Figure 9:**
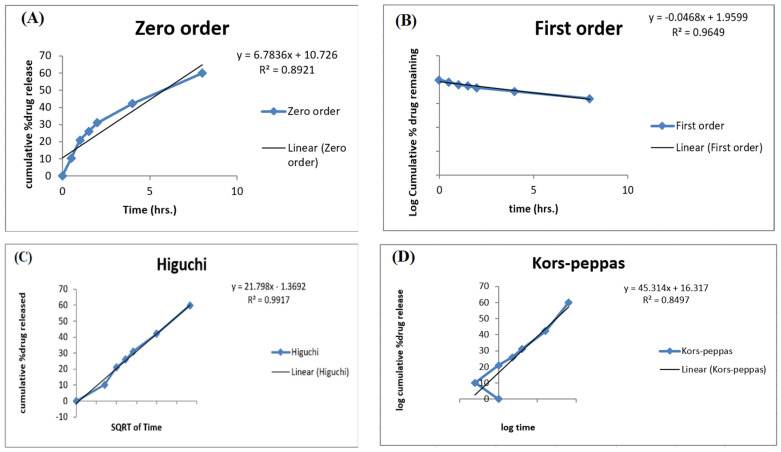
Different release kinetic models for optimized ACR-PCL-NPs. (**A**) zero-order, (**B**) first-order, (**C**) the Higuchi model, (**D**) the Korsmeyer-Peppas.

The release data of the optimized ACR-PCL-NPs were incorporated into different release models and evaluated for release mechanisms, as shown in Figure 9. To understand the process of drug release from nanoparticles, the data obtained were analyzed using four in vitro release models: zero-order, first-order, the Higuchi model, and the Korsmeyer–Peppas model.

In 1961, Higuchi proposed the Higuchi model to describe drug-release from matrix systems. The equation is:

(6) Qt=A√D(2C−Cs)Cst

where Qt is the amount of drug released from the united surface area “A” in time “t”. C is the initial drug concentration, Cs is the drug solubility in the matrix medium, and D is the drug diffusivity in a matrix [52].

The Higuchi model describes the drug release from a matrix as a diffusion process based on Fick’s law: when release kinetics are according to this model, the drug release from the particles will mainly be controlled by a diffusion process in a polymer matrix. To dig further into the mechanism of drug-release kinetics, the data obtained were analyzed using the Korsmeyer–Peppas model; the particles are considered spherical according to the morphological data [53]. The Korsmeyer–Peppas model is a semi-empirical model used for the analysis of different dosage forms’ release data, and the equation is given as follows:

(7) $$\frac{\mathrm{Mt}}{M\infty }={\mathrm{Kt}}^{n}$$

where Mt is the amount of drug at a time “t”, M∞ is the amount of drug at infinite time, K is the constant, and “n” is the release exponent.

To determine the n value in the Korsmeyer–Peppas model, only the Mt/M∞ ≤ 0.6 release fraction will be used. If the value of n is 0.43, it indicates that the dosage form releases the drug following Fickian diffusion. If the value is between 0.43 and 0.85, it will indicate the release of the drug by diffusion as well as by polymer chain relaxation [54]. In our case, release kinetics fitted the Higuchi model with an R^2^ value of 0.9917, which indicates that the active release of the drug from the dosage form was mainly controlled by a diffusion process. The data fitted in the Korsmeyer–Peppas model showed an R^2^ value of 0.8497 with an n value of 0.5285, which indicates drug release by two phenomena: drug diffusion and polymer chain relaxation. The Higuchi model is the perfect model to describe the drug dissolution profile of modified-release formulations, such as matrix tablets and transdermal systems with water-soluble drugs. The release- and kinetic-model studies were in agreement with the study of Miladi et al. [50].

### 3.4. Wound Re-Epithelialization

The percentage of the total epithelial covering formation on the wounded area at a specific time point is called % wound re-epithelialization. On day 1, the re-epithelialization of the wound was 0%. The rate of re-epithelialization on specific time points was greater in the formulation-treated group, as compared to the marketed drug-treated group and the control group. On day 3, the formulation drug-treated group had 17.14% ± 3.68% re-epithelialization, and the marketed drug-treated group and the control group had 10.12 ± 3.9% and 4.13% ± 2.11%, respectively (*p* < 0.001). On day 7, a significant difference was observed: the formulation drug-treated group was leading with 38.12% ± 8.15%, and the marketed drug-treated group and the control group had 31.10% ± 10.32% and 14.22% ± 15.54% re-epithelialization of a wound, respectively (*p* < 0.05). On day 14, the formulation drug-treated group achieved an almost-complete wound re-epithelialization (98.12% ± 0.32%), the marketed drug-treated group had 84.21% ± 3.12% (Table 5), and the control group had 65.22% ± 16.07% wound re-epithelialization (*p* < 0.001).

### 3.5. Tissue Growth and Gross Histology

**Figure 10:**
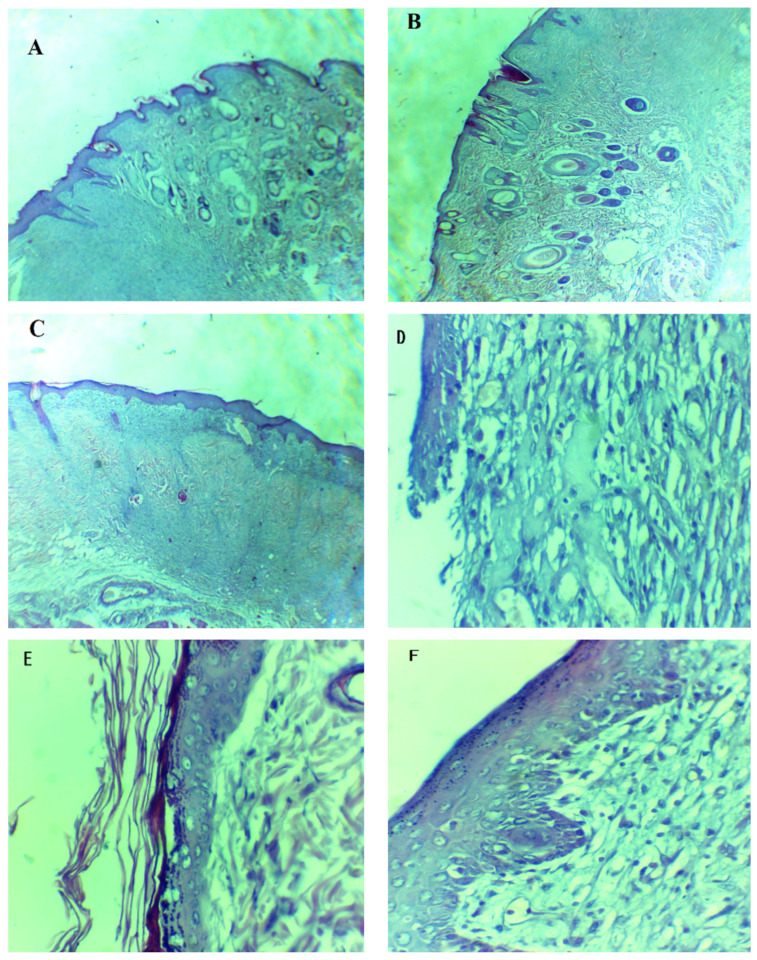
Hematoxylin- and eosin-stained burned epithelial tissues of rats for histopathological analysis. (**A**,**D**) are tissues from the control group, (**B**,**E**) are tissues from the marketed drug-treated group and (**C**,**F**) are tissues from the formulation-treated group. (**A**–**C**) = 5×, (**D**–**F**) = 40×.

The H- and E-stained slides of the controlled group, the marketed drug-treated group, and the formulation-treated group of rats were prepared for histological evaluation. The H- and E-stained slides were visualized and photographed using a light microscope at 5× and 40× magnification, which allowed us to visualize the recovered wound area and the formation of epidermis and dermis. The formulation-treated group showed an increase in burn-wound thickness, as compared to the marketed drug-treated and control groups. The formulation drug-treated group completed epidermal regrowth, with intact epidermises on the 14th day, while the marketed drug-treated group still had acute inflammatory infiltrations. The gap between the epidermis and dermis was small in the formulation-treated and marketed drug-treated groups, as compared to the control group. Blood vessels were smaller and higher in number in the formulation-treated group as compared to the marketed drug-treated group and the control group. The control group was much further behind in the regeneration process of the dermis and epidermis, and the persisting redness of the burn wound indicated inflammation and irregular granulation (Figure 10). The H and E staining study was also quantified by using a total skin-thickness measurement on a universal testing machine, showing an increase in the thickness of the formulation-treated group (1849 ± 240 µm, *p* < 0.01) and the marketed drug-treated group (1682 ± 340 µm, *p* < 0.05), compared to the untreated group (1152 ± 150µm). Hair follicles and rete pegs were not observed in the central wound, even though some were detected on the wound edges, which was likely due to the regeneration and migration of endogenous cells.

## 4. Conclusions

In this study, the developed optimized ACR-PCL-NPs showed desirable physicochemical properties. Nanoparticles were prepared using a double-emulsion solvent evaporation method, using PCL polymer for the encapsulation of the drug and polyvinyl alcohol as a stabilizing agent. A Box–Behnken design was applied to 15 formulations using Design Expert 11^®^. The developed ACR-PCL-NPs showed a particle size less than 780 nm, and a drug-entrapment efficiency of about 69.1%, with a spherical shape and a nearly neutral surface charge. The in vitro release was found to be biphasic, initially featuring a burst release for 1 h, followed by a sustained release for 24 h. The %age re-epithelization of the formulation drug-treated group (98.12 ± 0.32) was higher, as compared to the marketed drug-treated group (84.21 ± 3.12) and the untreated group (65.22 ± 16.07). Therefore, from all the above results, it can be concluded that optimized ACR-PCL-NPs could be a productive delivery system for acriflavine in burn-wound healing.

## Figures and Tables

**Table 1:** Independent and dependent variables.

Codes	Factors (Independent Variables)	
**X1**	Polymer concentration (gm)	
**X2**	Stirring speed (rpm)	
**X3**	Surfactant concentration (%)	
	**Responses (Dependent variables)**	
**Y1**	Particle size (nm)	
**Y2**	Polydispersity index (%)	
**Y3**	Entrapment efficiency (%)	
	**Formulation parameters which were kept constant**	
**Z1**	Dichloromethane	4 mL
**Z2**	Internal water phase volume (W1)	0.5 mL
**Z3**	External aqueous phase volume (W2)	60 mL
**Z4**	Primary emulsion stirring speed	3000 rpm for 3 min
**Z5**	Drug	20 mg
	Level of significance (α)	0.05

gm = gram; rpm = revolutions per minute; nm = nanometer; mL = milliliter; mg = milligram.

**Table 2:** Composition of the independent variables, 3 factors with 3 levels.

Factor Levels	Coded Values	Actual Values		
		Polymer concentration (X1)	Stirring speed (X2)	Surfactant concentration (X3)
Low	−1	1 g	8000 rpm	0.3%
Medium	0	1.5 g	10,000 rpm	0.5%
Maximum	+1	2 g	12,000 rpm	0.7%

**Table 3:** Design of experiment for the Box–Behnken method with 15 trial runs having actual values.

Runs	X1	X2	X3	Y1	Y2	Y3
F1	1	12,000	0.5	302.4	0.222	69.1
F2	1	10,000	0.3	378.6	0.251	67.7
F3	1	10,000	0.7	432	0.267	71.6
F4	1	8000	0.5	342.1	0.272	69.8
F5	1.5	12,000	0.7	499	0.266	70.1
F6	1.5	12,000	0.3	501.4	0.279	71.6
F7	1.5	10,000	0.5	463.2	0.288	75.9
F8	1.5	10,000	0.5	453	0.276	75.6
F9	1.5	10,000	0.5	471.2	0.301	75.2
F10	1.5	8000	0.3	596.2	0.372	74.6
F11	1.5	8000	0.7	670	0.352	74.1
F12	2	12,000	0.5	730	0.309	76.2
F13	2	10,000	0.3	710	0.399	78.9
F14	2	10,000	0.7	780.4	0.363	79.3
F15	2	8000	0.5	689.2	0.401	81.8

X1 = polymer concentration (g), X2 = stirring speed of external phase (rpm). X3 = surfactant concentration (%), Y1 = particle size (nm), Y2 = polydispersity index, Y3 = entrapment efficiency (%).

**Table 4:** Regression analysis summary for different models’ fitting data.

Model	R^2^	Adjusted R^2^	SD	Mean	% CV	*p*-Value
Response Y1						
Linear	0.8703	0.8350	61.35	534.58	10.42	0.0180
2FI	0.8802	0.7903	69.16	534.58	11.48	0.0130
Quadratic	0.9670	0.9076	45.91	534.58	8.59	0.0236
Response Y2						
Linear	0.9082	0.8832	0.0191	0.3078	6.19	0.3068
2FI	0.9348	0.8858	0.0189	0.3078	6.12	0.2950
Quadratic	0.9864	0.9619	0.0109	0.3078	3.54	0.6750
Response Y3						
Linear	0.8699	0.8344	1.66	74.10	2.24	0.0001
2FI	0.9097	0.8420	1.62	74.10	2.19	0.0037
Quadratic	0.9774	0.9368	1.03	74.10	1.39	0.0013

Y1 = particle size, Y2 = PDI, Y3 = entrapment efficiency, R^2^ = coefficient of correlation, SD = standard deviation, *p*-Value = associate probability values, CV = coefficient of variation.

**Table 5:** Rate of re-epithelialization of wounds at the 3rd, 7th, and 14th day (*n* = 6).

Days of Application of Dose	Wound Re-Epithelialization ± Standard Deviation (%)		
	Control Group	Marketed Drug-Treated Group	Formulation Drug-Treated Group
3	4.13 ± 2.11	10.12 ± 3.92	17.14 ± 3.68
7	14.22 ± 15.54	31.10 ± 10.32	38.12 ± 8.15
14	65.22 ± 16.07	84.21 ± 3.12	98.12 ± 0.32

## Data Availability

All data, models, and code generated or used during the study appear in the published article.
